# Seventy-five Hospitals at Army Camps Turned over to Surgeon General

**Published:** 1919-01

**Authors:** 


**Seventy-five Hospitals at Army Camps Turned Over to Surgeon General.**

---

Plans under which wounded men returning from France will go to hospitals within 300 miles of the homes of their nearest relatives have been announced by the War Department.

To this end base hospitals at training camps have been turned over to the Surgeon General, providing seventy-five hospitals with facilities to care for 104,231 men. Fifty thousand men are expected to be sent to these institutions within the next four months.

Use of the training camp facilities will make it possible to return to their owners immediately a number of properties which were to have been converted into general hospitals. These include the new Field Museum, Chicago; City Sanitarium, St. Louis, Mo.; Tulane University Buildings, New Orleans, and State School for the Blind, Nashville, Tenn.

The seventy-five hospitals to be employed do not include those at the ports of debarkation, New York and Newport News. There the wounded will be received on their arrival from France in fifteen institutions now ready for them with a bed capacity of 22,068. From the ports the men will be taken on specially fitted trains to one of sixteen localized hospital groups, where assignments will be made in such a way that each of the wounded men will go back to the region from which he entered the service. The groups include:

No. 12: General hospitals, Hot Springs and Fort Logan H. Roots, Ark., and base hospitals, Camps Pike, Beauregard and Shelby.

No. 14: General hospitals, Fort Bayard, N. M.; Whipple Barracks, Ariz., and Denver, Colo., and base hospitals, Camp Cody and Fort Riley.

No. 15: General hospital, Corpus Christi, Texas, and base hospitals, Camps Logan, Travis, MacArthur and Bowie, and at Forts Sam Houston, Sill and Bliss.

---

Make the "Almighty Dollar' to do the Lord's work by giving to the Armenian Relief Campaign, February 3-10.

